# Therapeutic potential of berberine against neurodegenerative diseases

**DOI:** 10.1007/s11427-015-4829-0

**Published:** 2015-03-06

**Authors:** WenXiao JIANG, ShiHua LI, XiaoJiang LI

**Affiliations:** 1 Department of Microbiology and Molecular Genetics, Emory University, Atlanta, GA 30322, USA; 2 Department of Human Genetics, Emory University, Atlanta, GA 30322, USA

**Keywords:** Huntington’s disease, berberine, autophagy, protein aggregation

## Abstract

Berberine (BBR) is an organic small molecule isolated from various plants that have been used in traditional Chinese medicine. Isolation of this compound was its induction into modern medicine, and its usefulness became quickly apparent as seen in its ability to combat bacterial diarrhea, type 2 diabetes, hypercholesterolemia, inflammation, heart diseases, and more. However, BBR’s effects on neurodegenerative diseases remained relatively unexplored until its ability to stunt Alzheimer’s disease (AD) progression was characterized. In this review, we will delve into the multi-faceted defensive capabilities and bio-molecular pathways of BBR against AD, Parkinson’s disease (PD), and trauma-induced neurodegeneration. The multiple effects of BBR, some of which enhance neuro-protective factors/pathways and others counteract targets that induce neurodegeneration, suggest that there are many more branches to the diverse capabilities of BBR that have yet to be uncovered. The promising results seen provide a convincing and substantial basis to support further scientific exploration and development of the therapeutic potential of BBR against neurodegenerative diseases.

Berberine (BBR) is an organic compound isolated from various herbs such as *Coptis chinensis*, *Berberis vulgaris*, *Hydrastis Canadensis*, *Phellodendron amurense.* For decades, Chinese medicine has used the plants and their extracts to treat diarrhea with no observable negative side-effects or toxicity in patients [1–3]. Modern advances in research, however, allowed us to discover BBR as the active compound and to synthesize it. As a result, BBR was found to be a small molecule with a molecular weight of only 371.8 Da [3] (Figure 1).

BBR has been used clinically to treat bacterial diarrhea, hypercholesterolemia, type 2 diabetes, cardiac disease, cancer, and more [3–14]. Although studies in rodents have shown that BBR can cross the blood brain barrier with positive effects on brain function, the mechanism remains unclear [15]. This factor points to the possibility that BBR may have pronounced effects on the brain and central nervous system. Additionally, in animal trials, BBR has shown itself to have positive effects on Alzheimer’s and Parkinson’s models [16,17]. Although we possess only a nascent understanding of BBR’s effects and mechanisms on the brain and nervous system, its protective effects on Alzheimer’s and Parkinson’s cellular and animal models and its uncanny ability to act with robust diversity, begin to shed light on BBR’s abilities to positively effect the outcome in diseases of the central nervous system.

According to the Center of Disease Control in the United States, an estimated 5 million people suffer from Alzheimer’s disease (AD) [18]. AD is a late-onset disease, typically presenting after age 60, and is characterized by memory loss and handicapped daily functions. To date, the exact cause of AD has not been pinpointed, with scientists currently believing the disease to arise from multiple contributing factors including genetic and environmental influences. However, pathological evidence always rests with beta-amyloid plaque build-up in the brain [19]. Despite the prevalence and severity of AD, its complexity has left modern science without answer, but in dire need of treatment options.

Parkinson’s disease (PD) is another common form of neurological disease that presents classically with resting tremor, rigidity, bradykinesia, postural instability and oftentimes, senile dementia [20]. PD is highly prominent especially in western European populations in which the prevalence rate is estimated at 160 per 100,000 and as high as 4% amongst persons over the age of 80 [20]. Although the direct pathological cause of PD has been stemmed to protein aggregations (called Lewy bodies) and loss of dopaminergic cells in the substantia nigra, the etiology behind PD is believed to be highly diverse [20,21] and the exact pathogenic mechanism is still unclear. Although current treatments for PD include dopamine agonists and monoamine oxidase B (MAO-B) inhibitors to reduce breakdown of dopamine, they are symptom-targeted and also produce serious side-effects [22]. To date, a cure or treatment without severe side-effects for PD has eluded modern science.

## 1 The therapeutic effect of BBR on AD

Although no single underlying cause has been established for beta-amyloid plagues-associated AD, metabolic imbalances have been found in AD patients and are likely to contribute to the symptoms of the disease. Due to its multi-faceted nature, BBR has been shown to address several of those imbalances in a positive way. These activities include cholesterol reduction, ERK pathway activation, inhibition of MAO-B activity, defense against damage from reactive oxygen species (ROS), inhibition of acetylcholinesterase (AChE), butyrylcholinesterase (BChE) activity, and beta-secretase, and reduction in the amyloid-beta genesis [3,23–26]. This has led to an overall reduction in amyloid plaque aggregation and reduction in phenotypic pathology of AD in the mouse model [27].

In 2009, Jung et al. [24] ran a number of inhibitory assays to determine the anti-AD effects of several protoberberine alkaloids. The IC50 (50% inhibitory concentration) was determined for each of the six compounds in their ability to stunt an AD-related activity. The inhibitory assays included β-site amyloid precursor protein cleaving enzyme 1 (BACE1), AChE, BChE, and reactive oxygen species (ROS). In addition to total ROS, peroxynitrite (ONOO^−^) scavenging was given particular attention due to its strong role in amyloid B formation. Through these assays, Jung et al. [24] found that although BBR was ineffective at inhibition of BACE1 and total ROS, the compound showed promising results against AChE, BChE, and ONOO^−^ (requiring 0.44, 3.44, and 23.06 μmol L^−1^ respectively to reach IC50 of a 100 μmol L^−1^ target solution).

In 2013, Panahi et al. [25] showed that with 50 mg kg^−1^ oral BBR taken daily, rabbits with chemically-induced AD-like symptoms showed improvement as compared to the untreated group. Although weight loss was only moderately abated, survival was significantly improved. Interestingly, the results showed significant inhibition of BACE1 in the treatment group, which stand in contrast to the prior findings of Jung et al. [24].

Asai et al. [26] also discovered that 10 μmol L^−1^ BBR could reduce amyloid B levels to 30% of the untreated amount *in vitro*. Furthermore, through Western-blotting of the different components of amyloid precursor protein (APP), they discovered that the mechanism behind the reduction was the result of APP pathway modulation towards a non-amyloid metabolite, as opposed to direct destruction of the protein itself or of its precursor [26].

Using a transgenic AD model mouse strain (CRN8D), Durairajan et al. [27] tested the effects of BBR on neurological impairment. The group found that BBR significantly reduced amyloid B plaque aggregation, leading to amelioration of mental and neuronal impairment in the treated group as compared to untreated transgenic control. Results also showed that these effects were achieved through inhibition of APP phosphorylation via suppression of glycogen synthase kinase (GSK) 3 activation [27]. In addition, the group found that treatment with a lower dosage (25 mg kg^−1^) often showed preferential results as compared to treatment with a higher dosage (100 mg kg^−1^) [27].

All these studies present consistent information of BBR’s effects against the various components of AD pathogenesis and its treatment results as manifested in the amelioration of AD pathology, presentation, and progression. Furthermore, the dosage study done by Durairajan et al. [27] suggests that to further develop the therapeutic capabilities of BBR against AD, biochemical approaches must be elicited, as increases in concentration can be counter-productive in treatment.

## 2 The therapeutic effect of BBR on PD

Although the pathology of Parkinson’s Disease (PD) has been pinned to the formation of Lewy body protein aggregates and loss of dopaminergic neurons in the brain, the causes that lead up to the pathology are largely unknown. Due to our lack of understanding of the true cause of this disease, effective treatment strategies remain to be developed. Thus, current therapies against PD are limited to amelioration of its symptoms, the main focus of which is stemmed around prevention or retardation of further dopaminergic neuronal loss. There has been substantially less research done on the therapeutic potential of BBR against PD than there has been against AD; mixed results and lack of clear success could be the main reasons. However, there are at least two documented effects that merit BBR or at least some derivative or metabolite in consideration for continued studies to further address the possibility of its use as an anti-PD agent.

In 2014, Kim et al. [28] used a chemically-induced mouse model of PD to test the neuro-protective effect of BBR. According to their data, at 50 mg kg^−1^, BBR significantly prevented both memory and balance loss in those PD mice as compared to their untreated counterparts. They found that there was corresponding lack of dopaminergic neuronal loss in the substantia nigra and decreases apoptosis in the hippocampus of the treated group as compared with the untreated control [28]. This recent study showed a very promising outlook for BBR for consideration as a select compound for treatment against PD.

However, the results of Kim et al. stand in stark contrast to the findings of the Lee group in South Korea, which published two accounts [29,30] of the neurotoxic effects of BBR when used to treat chemically-induced rat models of PD. In these studies, first, a PC12 cell model of PD showed that when treated with BBR, dopaminergic loss was elevated as compared to the untreated controls [29]. Treatment of a PD rat model with BBR (both at 5 and 15 mg kg^−1^) aggravated depletion of dopamine and norepinephrine as well as degeneration of tyrosine hydroxylase-immunopositive cells *in vivo* [30]. It is important to note that the differences in findings between the Lee group and Kim et al. could possibly be partially attributed to their differences in animal models (mouse vs. rat) and the species-dependent rates of BBR metabolism.

As MAO-A and MAO-B are both natural dopamine- degrading agents, their inhibition has always been therapeutic strategy against PD. However, due to the toxic side-effects of MAO inhibitors, this route has usually been reserved as the final effort in a patient’s treatment against advancing PD. Castillo et al. [31] used direct LED fluorescence to detect changes in MAO-B levels in hopes of using the method as a way to confirm the successes of various MAO-B inhibiting agents in PD treatment. Although it was previously known that BBR could inhibit MAO activity (with an IC50 of 126 μmol L^−1^ for MAO-A and 98.4 μmol L^−1^ for MAO-B), the results of this study put into perspective the usability of BBR as a MAO-inhibitor for PD treatment [23,31,32]. Compared to other MAO inhibitors that can often be highly toxic, BBR is highly preferable for its safety.

Bae et al. [33] discovered from *in vitro* studies using the human-derived SH-SY5Y cells as an *in vitro* model of dopaminergic neurons that BBR prevented cell death by protecting against ROS damage. The mechanism relied on activation of heme oxygenase-1 and inhibition of caspase-3 activation to parry neuronal apoptosis [33].

Taken together, while these results do show the possibilities of BBR as a therapeutic agent against PD, they also suggest proceeding with caution, as slight species-dependent differences in BBR metabolism could account for different efficacy or even effect of BBR.

## 3 General neuroprotective effects of BBR

BBR has also shown broadened neuroprotective effects. General oxidative damage to neurons and neurodegeneration plays an important role in a wide range of neurological disorders. To achieve its neuroprotective effects, BBR has been reported to activate nuclear factor-like 2 (Nrf2), aid in phosphorylation of Protein Kinase B (Akt), and cAMP response element binding protein (CREB), down-regulate nuclear factor kappa-light-chain-enhancer of activated B cells (NF-κB), and enhance phosphoinositide 3-kinase (PI3K) expression via (P55y subunit) increasing promoter activity [34–44].

Using mouse neuroblastoma cell lines, Hsu et al. [34] determined the *in vitro* neuroprotective effects of BBR. They found that in addition to the known anti-oxidative pathways, BBR was also capable of activating Nrf2, an anti-oxidant factor. Furthermore, they also found evidence that BBR stymied neuronal cell death by bolstering B-cell lym-phoma-2 (Bcl-2), an antagonist of apoptosis.

In addition, Zhang et al. [35] used a rat stroke model to test the abilities of BBR to protect against stroke. They found BBR to be neuroprotective in the acute phase of the stroke when administered immediately after insult. They also traced the BBR’s ability to up-regulate phosphorylation of neuroprotective factors: Akt, and CREB. BBR was also found to down-regulate the inflammatory response factor, NF-κB, to suppress inflammation in the area [35]. Interestingly, they also discovered that BBR can affect the permeability of the blood-brain barrier by up-regulating the integral membrane protein, claudin-5 [35].

In 2011, Hu et al. [36] found that in a mouse model with surgically induced stroke, BBR presented a unique way of bypassing a self-insulting down-regulation of the cell growth/proliferation factor, PI3K. In their model, BBR could target the P55y subunit of PI3K and enhance its promoter activity while side-stepping its natural antagonists, Ly294002 and Akti-1/2, which is also an Akt inhibitor [36]. This resulted in significantly enhanced reperfusion to the damaged areas as compared to the untreated controls [36]. Interestingly, this group chose caudal intravenous administration of BBR between 0.002 and 0.2 mg kg^−1^ as opposed to the more economic, safer, and much more commonly used oral gavage route.

## 4 Effects of BBR on the common pathways in neurodegeneration

**Figure 2:**
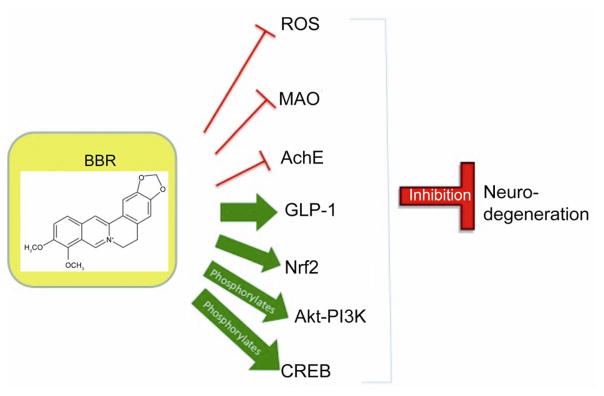
BBR interacts with many pathways that are affiliated with neurodegeneration or neuroprotection.

The multifaceted abilities of BBR to act against several neurodegenerative conditions or afflictions suggest that it may act on the shared pathways of neurodegeneration in these diseases. MAO-B inhibition has been implicated to have positive effects against both Alzheimer’s and Parkinson’s patients [45,46]. A possible reason could be that the MAO-B-mediated dopamine metabolism pathway leads to H_2_O_2_ generation and as MAO-B activity is elevated in AD and PD, the excess H_2_O_2_ can cause oxidative stress and damage [47]. Damage, as well as proapoptotic stimulation from reactive oxygen species (ROS) has also been implicated in a wide array of neurodegenerative afflictions [48,49] and the ability of BBR to counteract ROS certainly serves to uphold its ability to affect a diverse group of ailments in which neurodegeneration is a major factor. Furthermore, BBR’s inhibitory effects on AChE could lead to effects on a great number of neurodegenerative diseases as AChE has exhibited apoptotic induction, in addition to its general role as antagonist to acetylcholine [50]. Because of this, AChE inhibition is already in use as a therapeutic approach to curtailing neurodegeneration [51]. General neuro-protective factors that BBR can enhance or up-regulate include Nrf2, GLP-1 and others. Nrf2 has shown neuroprotection through a pathway that defends against ROS damage [52,53]. GLP-1 up-regulation and receptor stimulation has exhibited neuro-protective effects to preserve dopaminergic neurons, which have applications in stroke, AD, PD, Huntington’s disease (HD) and more [54–56]. Additionally, BBR’s ability to phosphorylate AKT and activate the PI3K-AKT signaling pathway results in the ability to resist apoptosis via inhibition of caspases and cytochrome C [57–59]. CREB is another important protein, the phosphorylation of which can be up-regulated by BBR. Research has shown pCREB to be an essential part of neurons that survive insult, and knocking it down could cause neurodegeneration in the brain [60,61]. Due to these common pathways that result in neurodegeneration, BBR is able to affect a great number of disorders in which neurodegeneration is key (Figure 2).

To summarize, BBR has exhibited promising neuroprotective properties through a multitude of known bio-molecular pathways and undoubtedly a vast number of unknown ones to not only defend against neuronal damage and loss but also to aid in reperfusion of previously damaged tissue. Furthermore, due to the multi-faceted nature of BBR, there are sure to be more that await discovery, some of which could hold the keys to some of the stubborn neurodegenerative diseases that need effective treatments.

## 5 Conclusion

BBR has shown great therapeutic potential against neurodegenerative diseases including AD and PD as well as stroke. Its ability to effectively cross the blood-brain barrier as well as its small size, which allows for the uncanny ability to act on a number of molecular targets, is a pillar of its merit as an anti-neurodegenerative agent. Because of the extensive range of effects, BBR is likely to treat diseases in a positive way by correcting multiple deficiencies and tackling the problem from multiple angles simultaneously. One possibility is that BBR may help clear toxic products or misfolded proteins to achieve multi-faceted benefits in reducing cellular toxicity. However, BBR is unlikely to have a very strong effect on any single one of these aspects, which may prevent harmful levels of imbalance and side effects. These facts coupled with its overall favorable effects on brain function suggest a potential myriad of other undiscovered applications on neurodegenerative diseases. The other pillar is its safety, as there have been no documented serious toxic effects even in high doses given orally. Because of this crucial feature, it has been safely clinically used in China for decades even for long-term treatment as an over-the-counter medication and is widely available in health stores in the US that require no prescription to purcase. Therefore, upon even mild-moderate success on animal models, BBR could quickly advance through clinical trials (for specific ailments) rather than taking the usual several long steps before being deemed safe. On a final note, the therapeutic potential of BBR shows promising potential and merits great interest and research investment for future development and exploration into the possibilities of this multi-faceted, non-toxic botanic compound.

## Figures and Tables

**Figure 1:**
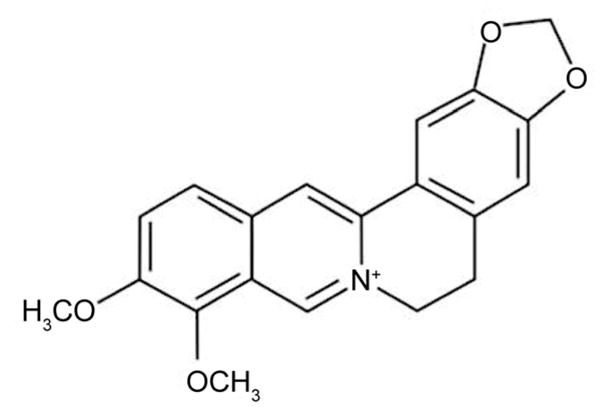
Molecular structure of berberine.
